# Quantitative Diet, Body Composition and Sprint Performance in Female Professional Beach Handball Players

**DOI:** 10.3390/nu15010138

**Published:** 2022-12-28

**Authors:** Alejandro Martínez-Rodríguez, Javier Sánchez-Sánchez, María Martínez-Olcina, Manuel Vicente-Martínez, Marcelo Peñaranda-Moraga, Nuria Asencio-Mas, Lucía Gonzálvez-Alvarado, Piotr Matlosz, Rodrigo Yáñez-Sepúlveda, Guillermo Cortés-Roco, Juan Antonio Sánchez-Sáez

**Affiliations:** 1 Department of Analytical Chemistry, Nutrition and Food Science, University of Alicante, 03690 Alicante, Spain; 2 Alicante Institute for Health and Biomedical Research (ISABIAL), 03010 Alicante, Spain; 3 School of Sport and Science, European University of Madrid, 28670 Madrid, Spain; 4 Faculty of Health Science, Miguel de Cervantes European University, 47012 Valladolid, Spain; 5 Institute of Physical Culture Sciences, Medical College of Rzeszów University, University of Rzeszow, 35-310 Rzeszów, Poland; 6 Faculty of Education and Social Sciences, Universidad Andres Bello, Viña del Mar 2520000, Chile; 7 Escuela de Educación, Pedagogía en Educación Física, Entrenador Deportivo, Universidad Viña del Mar, Viña del Mar 2520000, Chile; 8 Grupo de Investigación GDOT-Gestión Deportiva, Ocio y Tecnología, Faculty of Sport, Catholic University of Murcia, 30107 Murcia, Spain

**Keywords:** body composition, team sports, bone, somatotype

## Abstract

Women’s elite sports have experienced an exponential increase in the last decade, as has beach handball (BH). The high demands of this sport mean that athletes need to be in superior physical condition, so nutrition and body composition are determining factors in their sporting performance. For this reason, the aim of this study was to analyze, compare and correlate the most relevant variables of food intake (quantitative), body composition (focus on the bone mass characteristics) and sprint performance in female professional BH players. Thirty-three women from the National Spanish Team participated in this study. Dietary assessment, anthropometric measurements and sprint tests were performed. In general, the players had a low carbohydrate intake and adequate protein intake, with no significant differences depending on the category and playing position. For senior players, positive correlations were found between protein intake and bone mass (*r* = 0.584, *p* = 0.022), polyunsaturated fatty acid intake and muscle mass (*r* = 0.387, *p* = 0.026) and finally between fat mass and animal protein intake (*r* = 0.569, *p* = 0.027). Body composition was similar in both categories; however, goalkeepers had the highest fat (22.6 ± 3.86%, 16.2 ± 4.84 kg) component (vs. wings: 17.4 ± 3.53%, *p* = 0.031/vs. specialists: 11.1 ± 1.91 kg, *p* = 0.034), and senior players had higher muscle mass (kilograms). It is worth noting the finding that players with a greater trochanter height had significantly lower sprint times (*p* = 0.014 and *p* = 0.048 for 5 and 10 m, respectively). Certain bone characteristics, such as iliospinale height, biacromial and bimalleolar diameters, mesosternal perimeter and biceps skinfold, differ depending on the position. In addition, the greater speed of the senior players may be due to the greater specialization, number of training sessions performed and specific bone characteristics, such as trochanter height. In this regard, the data provided in this study will assist with establishing criteria for the selection of talent for this sporting discipline.

## 1. Introduction

Beach handball is characterized by motor characteristics such as accelerations, sprints or jumps, as well as rapid changes of direction and a high number of physical collisions [1,2]. The nutritional requirements of beach handball players, as seen in other team sports [3], will be variable throughout the season, affecting the intake of macronutrients, micronutrients and total energy. Currently, carbohydrate recommendations are between 5 and 12 g/kg body weight for moderate- to high-intensity exercise (duration of 1–4 h) [4], decreasing to 3–5 g/kg body weight for low-intensity exercise of up to one hour in duration [4]. Regarding proteins, with the objective of helping with muscle protein synthesis and recovery, recommendations of 1.2–2 g/kg body weight are established. As for micronutrients, female athletes have higher requirements, highlighting the importance of B vitamins [5], iron, calcium and vitamin D in athletes with a low caloric intake. The estimation of energy requirements in female beach handball players is a considerable challenge, as requirements may increase or decrease depending on age, general level of daily activity, training conditions and body composition [6]. 

In this sense, in the field of sport, the assessment of body composition is fundamental because it is one of the factors that can determine athletic potential and the probability of success in a particular sport, in combination with technical/tactical, physical, functional and psychosocial factors [7,8]. Body composition involves the analysis of the human body based on the fragmentation of total body mass. For beach handball, body fat should be monitored, as adequate fat levels allow players to move more efficiently during training and matches. Lean mass—in particular, muscle mass—should also be controlled, as inadequate training loads (excessive or insufficient) can lead to changes in physique which could affect performance factors such as speed, strength, power and injury risk [9,10].

In terms of bone mass, an article was recently published in which reference was made to the bone quality of these players [11]. It was observed that, after assessment of bone quality using a heel ultrasound densitometer, the broadband ultrasound attenuation (BUA) and speed of sound (SOS) values of female beach handball players were higher than those of both long- and short-distance runners [12,13,14], gymnasts [13] and powerlifters [15]. It seems, therefore, that the practice of this sport in the sand, as well as the repeated impacts after jumps, turns and sprints, favors the development and bone quality of the growing skeleton.

Bone is a highly dynamic tissue that adapts to changes in systemic signals, including hormones, as well as to mechanical stresses induced by physical activity [16]. Fatty acid intake is associated with increased bone mineral density, even reducing the risk of fracture [17]. In the case of female athletes, in order to maintain bone health, there are situations that may be relevant; including low energy availability, low carbohydrate availability, protein intake, vitamin D and dermal losses of calcium and sodium [18]. 

This highlights the critical role that properly planned and personalized nutrition plays in the bone health of female athletes and the necessity of a nutritional assessment of each athlete. This permits the determination of whether the required amounts of key nutrients are being consumed to support both bone health and optimal athletic performance. In fact, some morphological factors related to bone mass, such as leg length, have been found to regulate stride length, thus contributing to sprint performance [19]. Although previous studies described the anthropometric profile of beach handball [11,20,21] and handball players [22,23,24,25,26,27], none provided a complete and detailed description of all the bone diameters, lengths and body heights that are included in the complete ISAK (International Society for Advancement in Kinanthropometry) profile [28]. This is of particular interest to respond to different performance results, detect sporting talent and establish the athletic characteristics of athletes.

The main objective of this study was to describe and compare the dietary intake, anthropometrics, body composition, somatotypes and proportionality profiles of female professional beach handball players, according to age category (junior vs. senior) and playing position. Additional objectives were to correlate the different anthropometric variables with the results of the 5, 10 and 15 m sprints and provide data that can be useful in detecting sports talent. 

The hypothesis proposed is that senior players consume higher-quality diets since they are more aware of the importance of nutrition due to the length of time they have been practicing sports. It is also expected that protein consumption will be related to muscle mass, and high consumption of saturated fats to fat mass. In terms of body composition, senior female players will present a larger muscular compartment. Considering playing position, it is expected that goalkeepers will have the highest fat compartment. Speed results are expected to correlate with anthropometric characteristics rather than age category.

## 2. Materials and Methods

### 2.1. Study Design

A descriptive cross-sectional study was carried out to determine the body composition (fat mass, muscle mass, bone mass and residual mass, somatotype, body height and length, proportionality and speed) of female professional beach handball players. Due to their trajectory on the field, the players of the present research represent the world elite. The Declaration of Helsinki guidelines (revised in Fortaleza, Brazil, in October 2013) and the recommendations of Good Clinical Practice (Document 111/3976/88 of July 1990) were considered in carrying out the research. The ethics committee of the University of Alicante (Spain) (UA-2019-04-09) approved the study protocol.

### 2.2. Participants

A total of 33 players on the Spanish Beach Handball National Team participated in the research: 18 juniors and 15 seniors. These two national teams were champions and runners-up of the last IHF Women’s U18 Beach Handball World Championship (second edition) and the IHF Women’s Beach Handball World Championship (ninth edition) held in Greece in 2022; thus, they could be considered world top players. Considering the playing position, the sample was divided into 6 goalkeepers, 10 wings, 8 specialists, 6 pivots and 3 defenders. The exclusion criteria for this study included chronic disease, any injury that prevented the players from performing the established tests or not having completed the informed consent form. The players did not receive any economic compensation for their participation in the study. In the case of underage players, the parents or legal representatives were the ones who signed the consent form. The anonymity of the players was preserved throughout the research.

### 2.3. Data Collection

#### 2.3.1. Dietary Records 

For quantitative determination of players’ diets, they were asked to make a dietary record of four consecutive days (three during the week and one on the weekend) in the week prior to the intervention. The method of food weighing was chosen, since it is the method that offers the most information on quantity and frequency, and the record was accompanied by pictures of their meals. The players weighed what they ate and recorded all the foods they consumed during the established period, as well as the ingredients of the recipes or dishes consumed. They also had to indicate whether the food was raw or cooked [29,30,31]. The use of weighed food diaries has been suggested as the gold standard for assessing dietary intake in athletes [5].

#### 2.3.2. Anthropometric Measures

For the anthropometric evaluation, the guidelines established by the International Society for the Advancement of Kinanthropometry (ISAK) were followed [32]. Measurements were performed by an ISAK-accredited level 2 anthropometrist, considering within-subject technical measurement error (5% for skinfolds and 1% for circumferences, lengths and heights). All measurements were performed in the same location and under the same conditions (room temperature, 22 ± 1 °C). The 42 measurements included in the complete profile were obtained. Body mass or weight (kg) was measured by using a calibrated scale, Tanita, BC545N (Tokyo, Japan), with an accuracy of 100 g. For height (cm), a mobile anthropometer, Seca 213 (SECA Deutschland, Hamburg, Germany), with a precision of 1 mm, was used, keeping the heads of the female players in the Frankfort plane position. The 40 × 50 × 30 cm anthropometric bench was also used to measure sitting height. The distance between the points of the middle finger of the right and left hand was expressed in centimeters, and the wingspan was measured with an anthropometer that was placed on the wall and parallel to the floor.

The circumferences (head, arm (relaxed), arm (flexed and tensed), forearm (maximum), wrist (distal styloids), chest (mesosternal), waist, hip, thigh (1 cm gluteal), mid-thigh and calf) were measured with an inextensible metal tape measure. A Harpenden skinfold calliper (England; accuracy, 1 mm) was used for collection of the eight skinfolds (subscapular, tricipital, bicipital, iliac crest, supraspinal, abdominal, anterior thigh and medial leg). For small diameters (humerus, femur, wrist (bistyloid) and bimalleolar), a 22 cm pachymeter was used, and for large diameters (biacromial, bi-ilocristal, transverse chest and anterior–posterior chest depth), lengths and heights (acromiale–radiale, radiale–stylion, midstylion–dactylion, iliospinale height, trochanterion height, trochanterion–tibiale laterale, tibiale-lateral height, tibiale laterale–sphyrion tibiale and foot length), a 60 cm anthropometer and a Holtain segmometer (Holtain, Crymych, UK) were required. All the anthropometric instruments and equipment used were homologated and previously calibrated. 

Different formulas were used to calculate body composition: the Rocha [33] equation for bone mass and the Lee et al. (2000) [34] equation for muscle mass. Fat mass was calculated by using the Carter [35], Faulkner [36] and Withers + Siri formulas [37]. All variables were calculated in both percentages and kilograms. Residual mass was calculated from the difference between the total body weight and the sum of the bone, muscle and fat masses.

#### 2.3.3. Somatotype and Proportionality

The mean somatotype of each group of players (junior and senior) and of each playing position was calculated by following the method of Heath and Carter (1967) and classified according to the somatotype categories of Carter and Heath (1990). The phantom stratum was used for proportionality analysis [33]. 

Each variable was adjusted for phantom size, using the z-score. The z-values have a mean of 0, so a z-value of 0.0 indicates that the given variable is proportionally equal to the phantom; a z-value greater than 0.0 means that the subject is proportionally larger than the phantom for that variable; and, conversely, a z-value less than 0 shows that the subject is proportionally smaller than the phantom for the variable [33].

#### 2.3.4. Sprints 

The players performed sprints of 5, 10 and 15 m, starting from a ready position behind the starting line [38]. The sprint time was recorded by using two photocells (Witty Gate, Microgate, Mahopac, NY, USA) located at the starting line and at 5, 10 and 15 m, depending on the test to be recorded. All tests were performed on a sand surface, and the players were barefoot. The assessment started 15 min after the specific warm-up, based on Sánchez-Malia et al. [39]. The subjects first performed articular mobility exercises, and then they ran two 10 m races on a beach handball court, with the court measurements and sand characteristics according to the regulations of the International Federation. A standardized 10 min warm-up protocol was performed, consisting of different types of movement and five 10 m accelerations at a progressive intensity, with the last one at maximum speed. The rest period between warm-up repetitions was 1 min.

### 2.4. Statistical Analyses

Jamovi statistical software (version 1.6.15, The JAMOVI Project, Sydney, Australia) was used for data analysis. Descriptive calculations were performed for all variables included (mean and standard deviation), both overall and by age category and playing position. The Shapiro–Wilk test was used to test the normality of the distribution of descriptive variables. To evaluate the homogeneity of the data, the Levene test was used. To test for differences in basic measurements, body composition, somatotype, proportionality, anthropometric characteristics and sprints, both by category and by playing position, an analysis of variance (ANOVA), with a Bonferroni correction and a Tukey test, was applied. Statistical significance was set at *p* < 0.05. In addition, the effect size was calculated by using partial eta-squared (η^2^p), considering <0.25, 0.26–0.63 and >0.63 as small, medium and large effect sizes, respectively [40]. Partial omega squared (ω^2^) was also calculated in the case of the analyses by playing position, since the sample size of each group was smaller [41] (0.01–0.05, small effect; 0.06–0.13, moderate effect; and >0.14, large effect). An Excel template was used to obtain the z-phantom scores, which were represented as a graphic. The *t*-test was used to compare the differences in dietary intake between junior and senior players. Cohen’s d was used as a measure of the effect size (ES), considering small (d = 0.2), moderate (d = 0.6), large (d = 1.2), very large (d = 2.0) and extremely large (d = 4.0). Pearson’s correlation coefficient (*r*) was used to indicate how closely the variables were associated with each other. The relationship (or the correlation) between the variables was denoted by the letter “r” and quantified with a number, which varied between −1 and +1. A 0 means that there is no correlation, whereas a 1 means a complete or perfect correlation. The sign of the r shows the direction of the correlation. A negative r means that the variables are inversely related. The strength of the correlation increased both from 0 to +1 and from 0 to −1 [38].

## 3. Results

A total of 33 players (18 juniors and 15 seniors) of the Spanish Beach Handball National Team participated in the present study. Following quantitative evaluation of the players’ diets, as shown in Table 1, no significant differences were found between junior and senior players. Therefore, diet was not a confounding factor when analyzing the rest of the variables studied. However, if the results are compared with the dietary reference intakes (DRIs) for the Spanish population [42], the female players in the present study had lower intakes of most micronutrients than they should. When considering macronutrients [43], it seems that, in general, female players have a low carbohydrate (CH) intake compared to the recommendations, 5–7 g/kg/day. The same is true for proteins; it is estimated that sportswomen should consume between 1.6 and 1.8 g/kg/day, and the average coincided with these values. After the same analysis was performed by playing position, only one tendency (*p* = 0.052) was observed for the % CH variable; however, following the post hoc analyses, no significant differences were observed.

Regarding basic anthropometric measurements, the mean age was 16.7 ± 0.50 for the junior players and 24.8 ± 4.71 for the senior players. The descriptive data (mean ± standard deviation) and the ANOVA to test for differences between the basic measurements (wingspan, height, sitting height, weight and BMI), depending on the category, are shown in Table 2. There were no significant differences in any of the variables.

If these variables are analyzed according to playing position (goalkeepers, wings, specialists, pivots, and defenders), significant differences are observed in both wingspan and height (Table 3). There was a tendency (*p* = 0.053) for pivots to have a greater variable wingspan than wings. The same occurred for the height variable; however, in this case, the difference was significant (*p* = 0.014).

Table 4 and Table 5 show the following body composition variables: sum (∑) of six skinfolds (mm), ∑ of eight skinfolds (mm), fat mass with the Carter, Faulkner and Withers + Siri equations, both in percentage and kilograms, muscle mass (Lee et al. 2000 Equation), bone mass (Rocha’s Equation) and residual mass. Table 4, which shows the total statistics and those by age category, presents a significant difference (*p* = 0.013) in the variable kilograms of muscle mass, with the senior group reporting higher values (24.7 ± 1.83 kg) than the junior group (22.9 ± 1.96 kg).

Consideration of the playing positions changes the results. As shown in Table 5, there were significant differences in the variable fat mass (both in % and in kilograms) calculated with the Faulkner formula (*p* = 0.018 and *p* = 0.025, % and kilograms, respectively) and in the variable kilograms of bone mass (*p* = 0.049). Following the post hoc analyses, fat mass was observed to differ between goalkeepers and wings (*p* = 0.031) and between goalkeepers and specialists (*p* = 0.034), with fat mass being higher in goalkeepers in both cases.

For bone mass, following post hoc analysis, significant differences were observed between wings and pivots (*p* = 0.010) and between specialists and pivots (*p* = 0.021). The pivots had the highest bone mass (10.0 ± 0.627 kg), followed by the specialists (9.10 ± 0.653 kg) and the wings (9.03 ± 0.793 kg).

Following the correlation of these variables with dietary intakes (Table 1), in senior female players, positive correlations were observed between fat mass and animal-protein intake, both in % (*r* = 0.569; *p* = 0.027 and *r* = 0.552; *p* = 0.033 with the Faulkner and Withers formulas, respectively) and in kilograms (*r* = 0.590; *p* = 0.021 and *r* = 0.577; *p* = 0.024). Muscle mass (kg) was also positively correlated with grams of polyunsaturated fatty acids ingested (*r* = 0.636; *p* = 0.011). Bone mass (kg) was positively related with total grams of protein ingested (*r* = 0.593; *p* = 0.020), grams of protein/kg body weight and day (*r* = 0.584; *p* = 0.022) and monounsaturated fatty acids (*r* = 0.531; *p* = 0.042). No relevant correlations were found for junior players.

**Figure 1:**
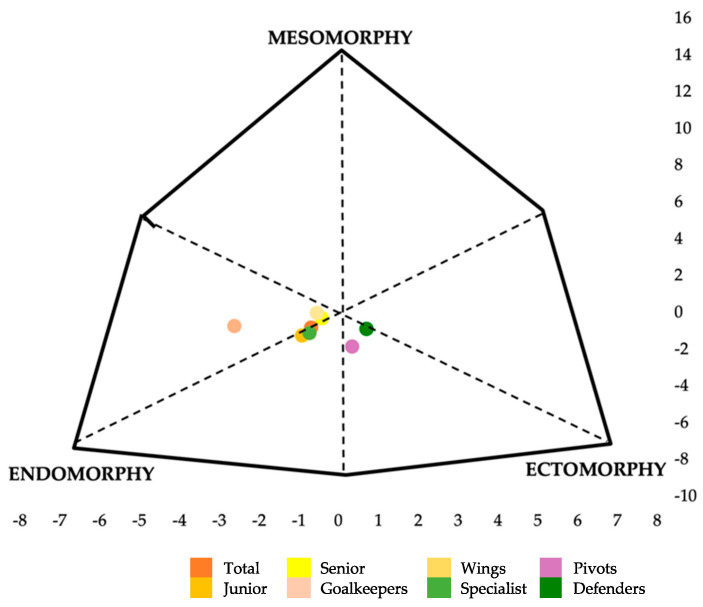
Somatotype distribution in elite female beach handball players.

Regarding somatotype, Figure 1 shows the mean somatotypes of the players by age category and playing position. There were no significant differences between the values obtained from junior and senior players in any of the three components: endomorphy (3.57 ± 1.12 vs. 3.16 ± 1.14, junior and senior, respectively), mesomorphy (2.48 ± 0.90 vs. 2.78 ± 0.951) and ectomorphy (2.66 ± 1.04 vs. 2.74 ± 1.17).

Regarding playing position, a significant difference was observed in the endomorphic component (*p* = 0.047). However, in the post hoc analysis with a Bonferroni correction, no significant differences were observed between any group. Following the analysis of this variable without correction, significant differences were observed between the position’s goalkeepers and wings (*p* = 0.014), goalkeepers and pivots (*p* = 0.013), and goalkeepers and defenders (*p* = 0.010), with endomorphy values decreasing in the following order: goalkeepers (4.55 ± 1.21) > wings (3.17 ± 1.14) > pivots (2.98 ± 0.88) > defenders (2.56 ± 0.75). 

**Figure 2:**
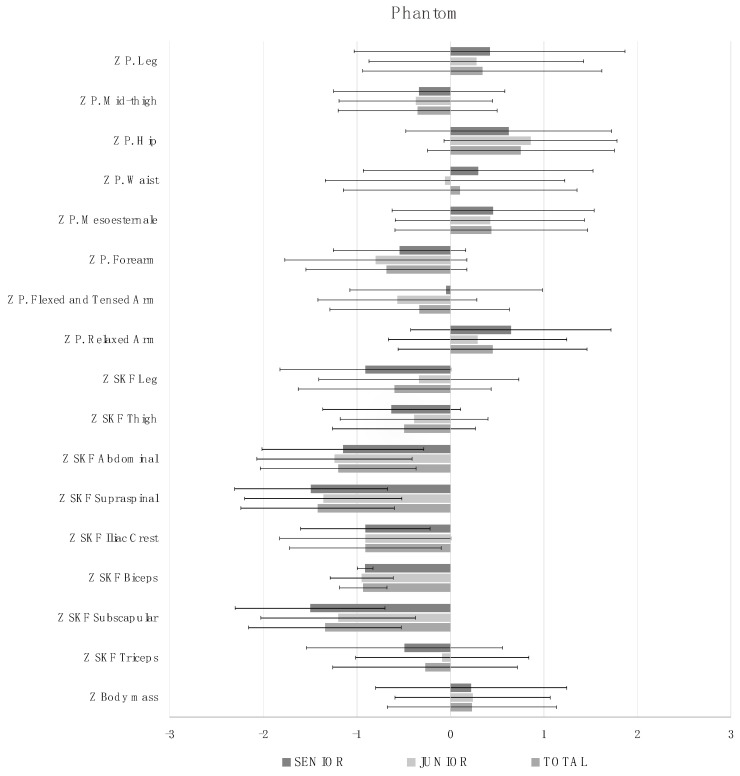
Proportionality with respect to the mannequin of the variables included in the ISAK restricted profile. SKF = skinfolds; P = perimeters.

The anthropometric dimensions and proportionality profile (of the 21 measurements that correspond to the restricted ISAK profile) of all the players and separated by age category are shown in Figure 2. After the analysis, no significant differences were observed in any of the variables.

In addition to skinfolds and perimeters, lengths, heights and large bone diameters were also collected. By age category, significant differences (*p* = 0.015) were only observed in the case of tibiale-laterale height, which was greater in senior players (46.3 ± 1.91) than in juniors (44.4 + 2.38). The descriptive data and differences for each of these variables, depending on the playing position, are shown in Table 6. Significant differences were observed in the variables iliospinale height (*p* = 0.032), biacromial diameter (*p* = 0.021), bimalleolar diameter (*p* = 0.025), mesosternal perimeter (*p* = 0.035) and hip perimeter (*p* = 0.014). Following the post hoc analyses, in the case of the iliospinale height, the female defenders had higher values than the female wings (*p* = 0.042). For biacromial and bimalleolar diameters, mesosternal perimeter and biceps skinfold, no significant differences were found after the post hoc analysis. Hip circumference was significantly higher in specialist players than in defenders (*p* = 0.023), and abdominal fold was significantly higher in goalkeepers than in wings (*p* = 0.040) and defenders (*p* = 0.016). 

**Figure 3:**
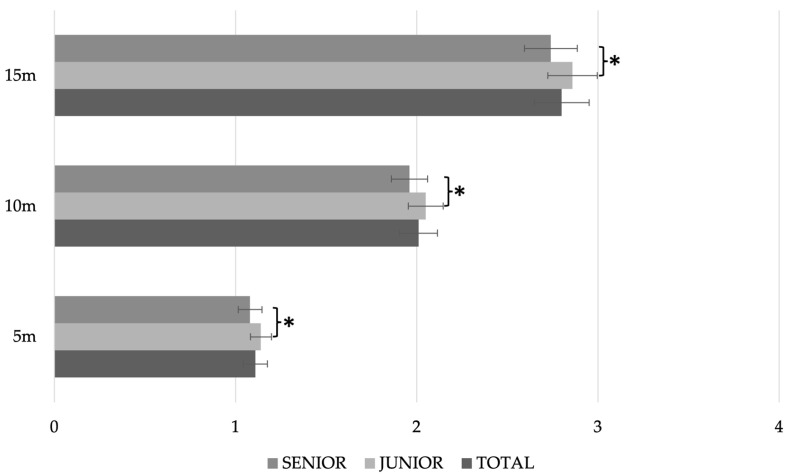
Sprint speed according to age category (junior vs. senior). * = significant differences *p* < 0.05.

**Figure 4:**
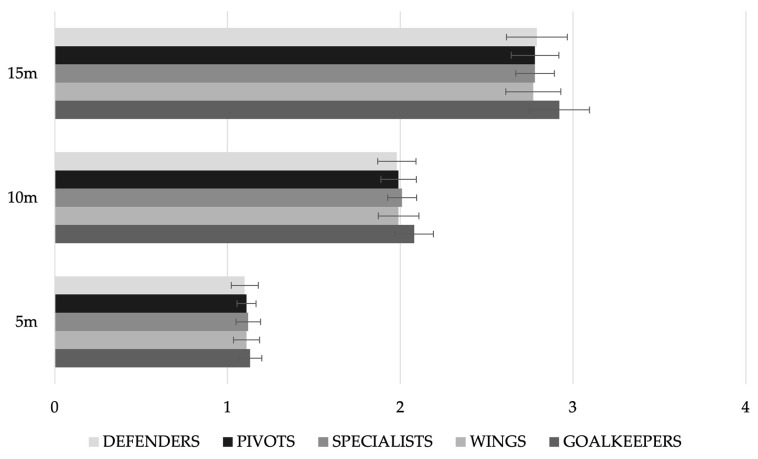
Sprint speed depending on playing position.

Figure 3 and Figure 4 show the results by age category and playing position, respectively. As can be seen in the figure, there were significant differences for the 5 m (*p* = 0.018), 10 m (0 = 0.020) and 15 m (*p* = 0.022) sprint, with the senior players being faster than the juniors in all cases. No significant differences were observed according to playing position (Figure 4). The results of a Pearson p-correlation analysis between the sprints and the different anthropometric variables showed significant positive correlations between the different tests of sprints and weight (*r* = 0.521; *p* = 0.002 and *r* = 0.576; *p* < 0.001 for 10 m and 15 m, respectively), transverse chest diameter (*r* = 0.390; *p* = 0.025 and *r* = 0.442; *p* = 0.010), neck perimeter (*r* = 0.390; *p* = 0.025 and (*r* = 0.420; *p* = 0.015), relaxed arm perimeter (*r* = 0.402; *p* = 0.020 and *r* = 0.427; *p* = 0.013), mesosternal perimeter (*r* = 0.452 and 0.455; *p* = 0.008 in both cases), waist (*r* = 0.445; *p* = 0.010 and *r* = 0.487; *p* = 0.004), hip (*r* = 0.586 and *r* = 0.582; *p* < 0.001 in both cases) and thigh (*r* = 0.463; *p* = 0.007 and *r* = 0.452; *p* = 0.008). 

The same occurred for the antero-posterior chest diameter (*r* = 0.426; *p* = 0.013, *r* = 0.510; *p* = 0.002 and *r* = 0.473; *p* = 0.005 for 5, 10 and 15 m, respectively), ∑ of six and eight skinfolds (mm), and % of fat mass (kg), with all the formulas used, in the case of 5 m *p* < 0.005 and for 10 and 15 m *p* < 0.001. It should be noted that there were also negative correlations between 5 m and 10 m sprints with trochanterion height (*r* = −0.423; *p* = 0.014 and *r* = −0.347; *p* = 0.048, respectively); therefore, those female players with a greater trochanterion height were faster. A similar pattern occurred with respect to % muscle mass and % bone mass. Significant negative correlations were observed with the 5 m sprint (*r* = −0.403 and *r* = −0.428 for muscle mass and bone mass, respectively; *p* = 0.013 in both cases), 10 m (*r* = –0.555; *p* < 0.001 and *r* = –0.529; *p* = 0.002) and 15 m (*r* = −0.586; *p* < 0.001 and *r* = −0.537; *p* = 0.001); therefore, the players with more muscle mass and more bone mass had faster results in the speed tests.

## 4. Discussion

The objectives of this research were to describe and compare the dietary, anthropometric, body composition, somatotype and proportionality profiles of professional female beach handball players according to age category (junior and senior) and playing position. Different anthropometric variables were also correlated with speed results (5, 10 and 15 m sprints).

Previous research has evaluated anthropometric characteristics, somatotype and body composition in beach handball players [9]; however, this study is the first to analyze the dietary intake of female players and to show the complete anthropometric profile of beach handball players as a function of playing position. These data were necessary, as this information could be of great help, together with fitness measurements, to determine physical preparation for competition and to monitor the effects of training and dietary interventions on body composition status and vice versa. However, in reference to the junior category, the players may be at different stages of development, and this should be taken into account to facilitate an effective transition of performance between the different categories (junior to senior) on the pathway to talent [44]. However, our analysis of the results indicated that the differences between categories (junior through senior) are not so great; therefore, these junior players may be close to the level of development of an elite senior female player.

One of the main findings following the quantitative dietary assessment was that carbohydrate intake was below the recommendations for players performing moderate training of approximately one hour per day (5–7 g/kg body weight) [4]: 3.33 ± 0.89 g/kg body weight in the case of juniors and 3.29 ± 0.797 in seniors. 

However, protein intake was indeed in line with current recommendations (1.2–2 g/kg body weight) [4], being 1.69 ± 0.36 and 1.69 ± 0.36 in juniors and seniors, respectively. Mean energy intake was 1684.85 ± 355.21 in juniors and 1701.39 ± 307.36 in seniors, lower than the 1870.46 ± 576.24 previously estimated in female soccer players [45] and 2073.13 and 2512.19 kilocalories observed in other team sports (soccer, tennis, basketball, football, golf, lacrosse, baseball and hockey) [46,47].

Considering that the mean intake of calcium and iron is 1000 mg/day and 18 mg/day, respectively, in both cases, the intake was lower: 595.26 ± 268.64 and 13.56 ± 5.16 mg/day in juniors and 613.64 ± 222.06 and 13.01 ± 4.24 mg/day in seniors, respectively. The vitamin D intake of the female players was 3.73 ± 6.02 and 4.35 ± 5.17 μg in juniors and seniors, complying with the current recommendations for vitamin D intake in female athletes, i.e., 5 μg [43]. The sodium intake of the players in the present investigation was slightly higher than the recommendations [43]. Considering the climatic conditions in which handball training sessions are held, this was to be expected, since 90% of the players reported consuming sports drinks before, during and after training. There is no concrete recommendation for sodium intake in female athletes, as there is some degree of interindividual variability, depending on sweat rates and individual sweat sodium concentrations [48].

In addition, in female athletes, menstruation, together with high-intensity training, can affect the status of other micronutrients, such as zinc and vitamin B12 [3]. The intakes of the players in the present investigation complied with both zinc (8 mg/day) and vitamin B12 (2 μg/day) recommendations, being 8.28 ± 2.36 and 4.69 ± 2.42 μg, respectively, in juniors and 10.68 ± 4.43 mg and 5.65 ± 2.33 μg, respectively, in seniors.

As for basic anthropometric measurements, the elite junior handball players showed a mean height of 167 ± 4.90 cm and a mean weight of 62.4 ± 7.29 kg, while in the senior players, these were 169 ± 5.31 cm and 64.9 ± 7.87 kg. As observed in male players [49], it seems that these differences are caused by the different age groups of players being in different stages of the maturation process. If these results are compared to those of the 32 senior players (25.3 ± 4.8 years) who were competing in the 2017 European Championship [1], both weight and height are slightly lower; 168 ± 3.86 cm and 60.78 ± 3.87 kg. In a study by Pueo et al. [9], both height 169.1 ± 5.1 and weight 62.9 ± 5.3 were more similar in the case of female players (24.1 ± 4.7 years).

Regarding these variables by playing position, an adequate comparison cannot be performed, since in the previous literature, players were grouped according to three playing positions [9]: goalkeepers (mesomorphic endomorph), wing-specialists (mesomorph-endomorph) and pivot-defenders (balanced ectomorph), with the latter two being categorized as the same position, despite their differences in specific training characteristics. However, similarly, the players in the present study had a balanced endomorphic anthropometric profile.

With the results obtained by playing position, it is possible to confirm the conclusion reached in a previous systematic review in female indoor handball players [10] that the weight and height values of wings are lower than those of other positions (Table 2). It seems that the reason for this finding is that wings need faster and lighter bodies, as they must be able to make rapid changes in speed and direction to cover as much of the field as possible. 

In the body-composition variables, significant differences (*p* = 0.013) were only observed in the variable muscle mass (kg), which was greater in the senior group (24.7 ± 1.83 kg) with respect to the junior group (22.9 ± 1.96 kg). However, after dividing the sample by playing position, significant differences were observed in fat mass calculated with the Faulkner formula, both in percentage (*p* = 0.018) and in kilograms (*p* = 0.025). Goalkeepers had significantly higher % fat mass (22.6 ± 3.86%) than wings (17.4 ± 3.53%) and greater fat mass (16.2 ± 4.84 kg) than specialists (11.1 ± 1.91 kg). This could be due to the goalkeepers’ own role in the team, as they move less than the other players and therefore have a lower energy expenditure. Differences were also observed in bone mass (*p* = 0.049); however, following the post hoc analysis with a Bonferroni correction, no significant differences were observed between positions. 

When the results of the present research were compared with those previously mentioned, it was affirmed that the players in the present research showed slightly higher fat mass, 18.9 ± 4.92%, compared to 15.4 ± 3.7% [9], calculated in both cases with the Withers + Siri formula. Following a correlation analysis of body composition variables with dietary intake, for senior female players, the consumption of animal protein was positively correlated with fat mass. The current evidence suggests that higher intakes of animal protein, i.e., in the quantities recommended for athletes, are unlikely to be adverse to bone health, assuming that the amount of calcium ingested is adequate [50]. However, it seems to have a negative effect on the accumulation of the fat-mass component, probably because the quality of the products chosen is inadequate, resulting in an increase in the intake of saturated fats [51]. In addition, correlations were found between bone mass (kg) and protein intake. This confirms previous findings [50], which suggested that protein may have an indirect effect on bone not only through its support of muscle mass and function but also through increasing circulating levels of insulin-like growth factor-1 (IGF-1), which has an anabolic effect on bone. 

When compared with those of female indoor handball players [10], the sum of six skinfolds (tricipital + subscapular + supraspinal + abdominal + thigh + leg) was slightly higher in female indoor players (93.81 mm vs. 84.5 mm). However, muscle mass was also greater in the case of the players on the national handball team [45], being 26.2 ± 2.6 kg, compared with 23.7 ± 2.07 kg in the players of the present investigation.

The female beach handball players in the present investigation showed a balanced endomorphic somatotype (endomorphy was dominant, while mesomorphy and ectomorphy were similar, not differing by more than 0.5), in both junior (3.6–2.5–2.7; endomorphy–mesomorphy–ectomorphy, respectively) and senior (3.2–2.8–2.7) players. These results do not coincide with those obtained by Pueo et al. [9], where the players presented an average mesomorphic–endomorphic somatotype (3.5–3.3–2.6). Although the endomorphy and ectomorphy components coincided, the mesomorphy of the players in the present study was slightly lower. However, the results are more similar to the results for indoor handball players (3.1–2.5–2.6) in a study by Marijana Cavala et al. [52]. This makes sense, since many of the players who play beach handball in the summer season are also indoor handball players during the winter. 

Nevertheless, this is not always the situation, as Ferragut et al. [45] observed, after analyzing the players of the Spanish national handball team, that the top elite women (26.4 ± 4.5 years) presented a slightly higher mesomorphic component than those reported previously (3.8–4.2–2.3). Regarding the playing position, although in the present research post hoc analyses with a Bonferroni correction did not show significant differences, the endomorphic component was higher in goalkeepers with respect to the rest of the positions: 4.55 ± 1.21 (goalkeepers), 3.17 ± 1.14 (wings), 3.40 ± 0.87 (specialists), 2.98 ± 0.88 (pivots) and 2.56 ± 0.75 (defenders).

Regarding proportionality, the profiles of both groups (junior vs. senior) were similar, with no significant differences in any of the variables. Because this is the first research showing a proportionality analysis in female beach handball players, it is not possible to compare our results with those of previous studies. The same is true for the variables large bone diameter, length and height. It is important to highlight the negative correlation between trochanterion height (vertical distance from the trochanterion mark to the ground) and the results of the 5 and 10 m sprints; a greater leg length was related to greater running speed. The same was true for muscle mass and bone mass; players with more muscle and bone mass were the fastest.

If these sprint results are compared with those of handball players from previous research [53], the players in the present study are faster (1.11 ± 0.07 and 2.01 ± 0.11 vs. 1.25 ± 0.06 and 2.19 ± 0.05 for 5 and 10 m, respectively). The same occurs in a comparison with beach handball players [54]; 1.17 ± 0.06 and 2.04 ± 0.08 s, for 5 and 10 m, respectively.

As in all research, reference should be made to the strengths and limitations presented. This is the first study to show the results of lengths, heights and proportionality of female beach handball players, contributing a complete data set to the literature. This provides useful data for recruiting and selecting players with an optimal anthropometric profile to improve performance and, consequently, team results. However, it is important to emphasize that, when comparing the results by playing position, caution should be exercised, since the groups did not have the same number of participants. It would be of great interest, in addition to obtaining the complete anthropometric profile, to use the gold-standard instrument DXA for the measurement of body composition. Although a quantitative analysis of the players’ intake was carried out, it would be necessary to carry out a qualitative analysis over a longer period, at least 7 days. In addition, the size of the sample and subsamples must be considered, so these data should be interpreted with caution. However, it should be recalled that beach handball is not considered a mass sport, despite its exponential growth in the last decade, especially in the women’s category, as is the case in other sports. However, in order to conduct this study, we had the opportunity to analyze the players of the Spanish Beach Handball National Team, i.e., world-class players, since in the last IHF Women’s Beach Handball World Championship—ninth edition—and in the IHF Women’s Youth Beach Handball World Championship (U18)—second edition—held in Greece in 2022, Spain won the silver medal and the gold medal, respectively. Therefore, the authors understand that the sample has a very exclusive value due to the difficulty accessing this type of population. Along these lines and for future research, players of different nationalities and a larger sample should be considered. Finally, it should be noted that this is a cross-sectional study, so it is not possible to establish causal relationships.

### Practical Implications

The present results can be used by coaches and fitness trainers in the creation of a high-performance plan for beach handball, as well as in the design of a talent discovery program. In this way, training can be specifically oriented to each type of female player, a fundamental aspect of elite performance.

## 5. Conclusions

Anthropometric characteristics, body composition and somatotype are important in team sports, including beach handball. In this study, the results are presented according to age category (junior vs. senior) and the six playing positions (goalkeepers, wingers, specialists, pivots and defenders). The intake of protein and some micronutrients, such as sodium, vitamin D, zinc and vitamin B12, were adequate in beach handball players. However, the intake of carbohydrates, calcium and iron did not meet the recommendations. In the basic measures, there were no significant differences between junior vs. senior players; however, there were significant differences in terms of playing position for the height variable, which was significantly higher in pivots than in wings. Body composition was similar in both groups; however, it should be noted that female goalkeepers had the highest fat component. Regarding somatotype, in both categories, the somatotype was balanced endomorph. An important finding of this research was the correlation between trochanter height and faster results for sprint speed. These data enrich the literature published so far, offering a reference for female beach handball players.

## Figures and Tables

**Table 1:** Quantitative analysis of the players’ diets.

	Junior (*n* = 18)		Senior (*n* = 15)		*t*-Test		
	Mean	SD	Mean	SD	*p*	MD	ES
Energy (kcal)	1684.85	355.219	1701.39	307.361	0.888	−165.433	−0.0495
Energy (kcal/kg/day)	31.81	6.694	32.11	5.794	0.890	−0.308	−0.0488
Carbohydrates (g/kg/day)	3.33	0.894	3.29	0.797	0.877	0.047	0.0548
Protein (g/kg/day)	1.69	0.358	1.81	0.389	0.346	−0.124	−0.3343
Sodium (mg)	1975.23	703.609	1922.95	700.834	0.833	522.867	0.0744
Cholesterol (mg)	289.03	97.276	362.31	116.765	0.058	−732.733	−0.6879
Carbohydrate (g)	176.24	47.665	173.87	41.358	0.881	23.711	0.0528
Protein (g)	89.12	19.469	95.79	20.898	0.350	−66.767	−0.3317
Lipids (g)	69.45	18.803	68.98	13.875	0.937	0.470	0.0280
Fiber (g)	20.33	8.448	20.18	5.809	0.953	0.153	0.0208
Potassium (mg)	2714.18	913.604	2846.20	668.261	0.645	−1.320.167	−0.1626
Calcium (mg)	595.26	268.639	613.64	222.063	0.834	−183.789	−0.0739
Magnesium (mg)	277.12	104.975	279.42	80.273	0.945	−22.978	−0.0243
Phosphorus (mg)	1120.11	278.005	1221.46	292.407	0.316	−1.013.544	−0.3561
Iron (mg)	13.56	5.164	13.01	4.246	0.744	0.549	0.1150
Selenium (mg)	71.34	26.352	83.03	27.562	0.223	−116.944	−0.4347
Zinc (mg)	8.28	2.363	10.68	4.429	0.056	−23.967	−0.6941
Vitamin B12 (μg)	4.69	2.422	5.65	2.326	0.258	−0.959	−0.4031
Folate (μg)	242.19	82.022	263.42	74.447	0.446	−212.256	−0.2697
Vitamin D (μg)	3.73	6.019	4.35	5.174	0.756	−0.619	−0.1095
Carbohydrates (%)	41.74	7.007	40.77	4.977	0.656	0.971	0.1573
Protein (%)	21.34	3.012	22.49	2.482	0.247	−11.489	−0.4125
Lipids (%)	36.99	6.115	36.60	4.813	0.843	0.389	0.0699

kcal = kilocalories; kg = kilograms; mg = milligrams; g = grams; μg = micrograms; % = percentage; SD = standard deviation; MD = mean differences; ES = effect size.

**Table 2:** Descriptive data and differences in basic measures according to category.

	Total (*n* = 33)		Junior (*n* = 18)		Senior (*n* = 15)		ANOVA			
	Mean	SD	Mean	SD	Mean	SD	F	*p*	η^2^p	ω^2^
Wingspan (cm)	171	5.94	170	6.23	172	5.52	1.14	0.295	0.035	0.004
Height (cm)	168	5.15	167	4.90	169	5.31	1.73	0.198	0.053	0.022
Sitting height (cm)	88.2	2.53	88.3	2.76	88.1	2.31	0.07	0.787	0.002	−0.029
Weight (kg)	63.6	7.54	62.4	7.29	64.9	7.87	0.85	0.361	0.027	−0.004
BMI (kg/m^2^)	22.6	2.47	22.5	2.28	22.8	2.75	0.08	0.767	0.003	−0.028

*n* = number per group; SD = standard deviation; cm = centimeters; kg = kilograms; m = meters; BMI = body mass index; η^2^p = partial eta-squared; ω^2^ = omega squared.

**Table 3:** Descriptive data and differences in basic measures according to playing position.

	Goalkeepers (*n* = 6)		Wings (*n* = 10)		Specialists (*n* = 8)		Pivots (*n* = 6)		Defenders (*n* = 3)		ANOVA			
	Mean	SD	Mean	SD	Mean	SD	Mean	SD	Mean	SD	F	*p*	η^2^p	ω^2^
Wingspan (cm)	174	6.15	168 *	3.41	168	4.92	175 *	5.58	175	7.18	3.93	0.012	0.359	0.262
Height (cm)	169	3.72	164 *	3.58	166	4.89	172 *	3.96	172	6.60	4.46	0.006	0.389	0.295
Sitting height (cm)	88.7	2.57	87.2	2.92	87.6	2.51	89.1	0.971	90.5	2.14	1.55	0.216	0.181	0.062
Weight (kg)	70.8	9.30	60.2	8.07	61.2	4.12	64.4	4.38	64.8	7.12	2.55	0.062	0.267	0.158
BMI (kg/m^2^)	24.9	3.60	22.2	2.50	22.3	1.45	21.8	1.72	21.8	1.31	1.85	0.148	0.209	0.093

*n* = number per group; SD = standard deviation; cm = centimeters; kg = kilograms; m = meters; BMI = body mass index; η^2^p = partial eta-squared; ω^2^ = omega squared; * = significant differences *p* < 0.05.

**Table 4:** Skinfolds and body composition by age category.

	Total (*n* = 33)		Junior (*n* = 18)		Senior (*n* = 15)		ANOVA		
	Mean	SD	Mean	SD	Mean	SD	F	*p*	η^2^p
∑ 8 Skinfolds (mm)	84.5	25.6	87.5	26.2	80.8	25.3	0.557	0.461	0.018
∑ 6 Skinfolds (mm)	106	30.4	109	31.9	103	29.1	0.346	0.561	0.011
FM Carter (%)	16.7	3.96	17.1	4.05	16.1	3.92	0.557	0.461	0.018
FM Faulkner (%)	18.3	3.64	18.6	3.66	18.0	3.71	0.175	0.679	0.006
FM Faulkner (kg)	11.9	3.75	11.8	3.67	11.9	3.96	0.012	0.911	0.000
FM Withers + Siri (%)	20.4	4.91	21.6	4.70	18.9	4.92	2.540	0.121	0.076
FM Withers + Siri (kg)	13.2	4.70	13.8	4.66	12.6	4.84	0.490	0.489	0.016
MM Lee 2000 (kg)	23.7	2.07	22.9 *	1.96	24.7 *	1.83	6.920	0.013	0.182
MM Lee 2000 (%)	37.6	3.92	36.9	2.72	38.4	4.97	1.280	0.266	0.040
Bone Mass (kg)	9.42	0.796	9.32	0.897	9.55	0.661	0.714	0.404	0.023
Bone Mass (%)	14.9	1.26	15.0	1.19	14.9	1.37	0.096	0.759	0.003
Residual Mass (kg)	17.1	2.37	16.4	1.36	18.0	3.02	4.060	0.053	0.116
Residual Mass (%)	27.0	2.56	26.4	2.09	27.7	2.96	1.960	0.172	0.059

*n* = number per group; SD = standard deviation; mm = millimeters; kg = kilograms; % = percentage; FM = fat mass; ∑ = summatory; MM = muscle mass; η^2^p = partial eta-squared; ω^2^ = omega squared; * = significant differences *p* < 0.05.

**Table 5:** Skinfolds and body composition according to playing position.

	Goalkeepers (*n* = 6)		Wings (*n* = 10)		Specialists (*n* = 8)		Pivots (*n* = 6)		Defenders (*n* = 3)		ANOVA			
	Mean	SD	Mean	SD	Mean	SD	Mean	SD	Mean	SD	F	*p*	η^2^p	ω^2^
∑ 8 Skinfolds (mm)	111	25.1	80.2	29.1	81.0	14.0	78.7	25.4	67.0	7.19	2.50	0.065	0.263	0.154
∑ 6 Skinfolds (mm)	137	28.1	101	34.9	104	18.9	98.3	29.9	86.3	9.26	2.36	0.078	0.252	0.142
FM Carter (%)	20.7	3.88	16.0	4.50	16.1	2.16	15.8	3.93	14.0	1.11	2.50	0.065	0.263	0.154
FM Faulkner (%)	22.6	3.86	17.4	3.53	18.0	2.56	17.3	2.93	15.9	1.51	3.58	0.018	0.338	0.238
FM Faulkner (kg)	16.2	4.84	10.7	3.77	11.1	1.91	11.2	2.34	10.4	2.04	3.27	0.025	0.319	0.216
FM Withers + Siri (%)	24.6	5.87	19.6	5.95	20.1	2.91	19.3	3.81	17.6	1.83	1.59	0.204	0.185	0.067
FM Withers + Siri (kg)	17.8	6.44	12.2	5.42	12.4	2.26	12.5	2.79	11.4	2.11	1.93	0.133	0.216	0.101
MM Lee 2000 (kg)	24.7	2.84	23.3	2.06	22.4	1.09	24.1	1.85	25.6	0.653	2.14	0.102	0.234	0.121
MM Lee 2000 (%)	35.0	2.60	39.3	5.78	36.7	1.24	37.5	2.20	39.8	3.98	1.59	0.206	0.185	0.066
Bone Mass (kg)	9.71	0.806	9.03	0.793	9.10	0.653	10.0	0.627	9.79	0.599	2.72	0.049	0.280	0.173
Bone Mass (%)	13.8	1.29	15.1	1.23	14.9	1.16	15.6	1.06	15.2	1.23	1.91	0.137	0.214	0.099
Residual Mass (kg)	18.6	1.44	15.6	2.53	17.2	1.62	17.7	1.48	17.9	4.51	2.14	0.102	0.234	0.121
Residual Mass (%)	26.6	3.04	25.9	2.86	28.2	1.65	27.6	1.72	27.4	3.81	1.04	0.406	0.129	0.004

*n* = number per group; SD = standard deviation; mm = millimeters; kg = kilograms; % = percentage; FM = fat mass; ∑ = summatory; MM = muscle mass; η^2^p = partial eta-squared; ω^2^ = omega squared.

**Table 6:** Lengths, heights, diameters, perimeters and skinfolds depending on the playing position.

	Goalkeepers (*n* = 6)		Wings (*n* = 10)		Specialists (*n* = 8)		Pivots (*n* = 6)		Defenders (*n* = 3)		ANOVA		
	Mean	SD	Mean	SD	Mean	SD	Mean	SD	Mean	SD	F	df2	*p*
L Acromiale–Radiale	32.5	1.91	30.9	1.12	31.9	0.975	33.1	1.12	33.1	1.82	3.526	9.18	0.053
L Radiale–Stylion	25.2	1.84	24.1	0.693	24.1	1.03	24.9	1.54	24.8	1.37	0.859	8.80	0.524
L Stylion Medio-Dactylion	19.5	0.632	19.1	0.717	19.0	0.824	19.8	0.771	19.6	0.100	2.113	12.95	0.137
H Iliospinale	95.0	3.08	91.2	1.73	92.0	3.45	98.0	4.05	95.1	4.86	4.380	8.67	0.032
H Trochanterion	90.8	5.08	89.7	4.91	88.0	5.27	92.4	4.76	88.7	2.53	0.733	11.28	0.588
H Trochanterion–Tibiale Laterale	43.3	1.59	42.3	1.54	42.0	1.79	44.9	2.74	42.7	1.44	1.372	9.89	0.312
H Tibiale Laterale	46.1	1.49	44.1	1.60	44.3	2.22	47.2	2.37	46.0	4.10	2.512	9.12	0.115
L Foot	25.8	0.912	24.9	0.810	25.1	0.811	26.1	1.27	25.9	0.971	1.695	9.47	0.231
L Tibialis Mediale–Sphyrion Tibiale	39.7	1.64	37.2	1.45	37.1	1.39	39.5	2.61	40.0	2.93	3.275	9.05	0.064
D Biacromial	37.7	1.97	36.1	0.867	36.1	1.59	34.8	4.26	39.5	1.18	5.012	9.08	0.021
D Antero-Posterior Abdomen	20.3	1.96	17.6	1.56	17.4	1.34	17.4	1.57	18.1	2.45	2.175	9.26	0.151
D Bi-Iliocristal	29.1	1.79	26.4	2.07	27.1	1.02	29.4	3.70	27.5	0.819	2.157	10.79	0.143
D Transverse Thoracic	31.8	3.72	28.6	1.05	28.4	0.952	28.4	1.10	29.4	0.961	1.436	9.73	0.294
D Antero-Posterior Thorax	16.9	1.91	15.4	1.62	15.7	1.43	15.4	1.14	15.5	0.737	0.694	11.36	0.611
D Humerus	6.23	0.294	6.11	0.256	6.24	0.200	6.40	0.261	6.30	0.200	1.063	9.97	0.424
D Bistyloid	4.93	0.242	4.91	0.185	4.86	0.226	5.08	0.147	5.03	0.057	1.877	12.75	0.176
D Femur	9.17	0.513	8.76	0.610	8.79	0.181	8.97	0.333	8.73	0.115	1.208	11.75	0.359
D Bimalleolar	6.83	0.339	6.50	0.226	6.66	0.213	6.88	0.240	7.07	0.208	4.518	9.82	0.025
P. Head	56.5	3.57	55.0	2.16	54.9	0.888	54.8	1.07	54.1	1.42	0.479	9.47	0.751
P. Neck	33.0	1.97	31.3	1.91	31.7	1.63	32.2	1.00	32.2	0.462	0.762	12.88	0.568
P. Relaxed Arm	29.3	3.06	27.1	2.29	26.7	1.94	27.3	1.82	27.7	0.153	1.030	12.49	0.430
P. Arm Flex Contra	30.2	2.82	27.9	1.69	27.1	2.10	27.8	2.28	28.8	1.02	1.385	11.00	0.301
P. Forearm	24.5	1.66	23.4	1.31	23.7	0.793	23.7	1.24	24.3	0.987	0.667	9.71	0.630
P. Wrist	14.8	0.625	15.4	3.08	14.4	0.431	14.8	0.210	14.7	0.200	1.510	11.43	0.263
P. Mesoternal	93.5	5.39	86.6	3.61	88.5	4.27	87.0	2.65	90.0	0.751	3.583	13.16	0.035
P. Waist	76.4	6.64	69.1	5.40	70.7	4.36	70.3	1.87	71.5	4.45	1.165	9.47	0.385
P. Hip	103	6.11	95.2	6.72	95.0	2.94	97.1	2.90	99.9	1.22	4.821	12.71	0.014
P. Thigh 1 cm	61.0	5.43	55.7	3.40	59.9	15.3	54.9	2.45	57.6	1.66	1.938	11.66	0.170
P. Mid-Thigh	54.4	4.77	49.9	3.77	49.3	2.18	50.3	3.48	51.5	0.551	2.438	13.03	0.099
P. Leg	36.7	2.97	35.7	3.42	33.9	1.86	35.6	2.36	36.1	0.586	2.176	13.08	0.129
P. Ankle	21.7	1.97	21.1	1.06	20.9	1.15	21.3	0.819	21.1	0.603	0.213	10.93	0.925
SKF Triceps	18.3	5.52	13.3	4.84	13.5	1.51	13.0	3.57	10.8	2.39	1.733	9.30	0.224
SKF Subscapular	14.5	4.73	9.43	3.85	10.0	3.54	8.63	1.78	8.33	2.45	1.857	10.14	0.194
SKF Biceps	8.98	3.36	6.44	3.97	6.14	1.80	6.91	1.56	4.58	0.749	3.634	12.62	0.035
SKF Iliac Crest	19.4	5.10	15.1	5.94	17.1	5.20	13.8	5.01	13.1	2.91	1.500	11.16	0.268
SKF Supraspinal	12.3	3.51	7.93	3.72	8.99	2.88	8.08	3.47	6.83	2.25	1.926	10.50	0.179
SKF Abdominal	23.9	5.79	13.9	5.39	15.0	4.80	14.2	6.15	11.8	1.72	4.930	12.53	0.013
SKF Thigh	27.2	3.35	22.6	7.81	21.2	3.78	21.0	8.03	19.0	3.66	3.227	10.24	0.059
SKF Leg	14.5	4.68	13.0	6.45	12.3	2.87	13.8	5.09	10.3	2.25	0.896	10.94	0.499

SD = standard deviation; L = length; H = height; D= diameter; P = circumference; SKF = Skinfold; df2 = degrees of freedom 2Regarding sprint speed.

## Data Availability

The data presented in this study are available upon request from the corresponding author. The data are not publicly available due their containing personal health information.
